# Laser Spectroscopic Sensors for the Development of Anthropomorphic Robot Sensitivity

**DOI:** 10.3390/s18061680

**Published:** 2018-05-23

**Authors:** Oleg Bukin, Dmitriy Proschenko, Alexey Chekhlenok, Sergey Golik, Ilya Bukin, Alexander Mayor, Victoriya Yurchik

**Affiliations:** 1 Maritime State University Named after G.I. Nevelskoy, Verkhneportovaya 50A St., 690059 Vladivostok, Russia; dima.prsk@mail.ru (D.P.); publicarkbenefit@gmail.com (A.C.); il_bukin@mail.ru (I.B.); vita_yurchik@mail.ru (V.Y.); 2 Far Eastern Federal University, Sukhanova 8 St., 690091 Vladivostok, Russia; golik_s@mail.ru (S.G.); mayor@iacp.dvo.ru (A.M.); 3 Institute of Automation and Control Processes FEBRAS, Radio 5 St., 690041 Vladivostok, Russia

**Keywords:** laser induced fluorescence, laser induced breakdown spectroscopy, limit of detection, sensors, underwater robotics, artificial hand, femtosecond laser pulse, oil, seawater

## Abstract

The development of underwater robotics sensitivity, which is based on the sensors of laser spectroscopy methods, have been discussed. The ways to improve Laser Induced Fluorescence (LIF) and Laser Induced Breakdown Spectroscopy (LIBS) methods were investigated in order to develop and create laser sensitivity for underwater robotics. A brief overview is done in the article, where LIF and LIBS spectroscopy in underwater robotics are used as spectroscopy sensors in order to investigate underwater environments by means of underwater vehicles. Limit of Detection (LoD) of oil and oil product solutions in the seawater have been detected by means of nanosecond and femtosecond spectroscopy LIF. All results, which had been received by laser pulses of different duration, were compared. The same experiments have been provided in order to measure concentrations of elements in the seawater and solutions by the LIBS method. It was discovered that the LoD of a group of elements was reduced when the femtosecond LIBS was used. Anthropomorphic complexes were under discussion in order to adopt laser spectroscopy sensors for underwater environments. The submersible module, which was constructed to investigate and examine laser spectroscopy sensors, has been described.

## 1. Introduction

The development of sensors for underwater robots is an exciting prospect. Underwater vehicles are used in extreme environmental conditions, so it is very important to equip underwater robots with sensing devices. Today there are sensors specifically developed to provide the exact functions necessary to sense an underwater environment. Acoustic sensors are widely used devices that locate and measure distance, survey a bottom, observe underwater objects, and measure the coordinates of underwater vehicles. If acoustic and optic sensors are compared, the sense of “hearing” is better than a sense of “vision” in underwater robotics. In spite of this fact laser sensors are currently under development that can fully complete data from acoustic sensors. Laser sensors for the range, size measurement, and form identification of underwater objects provide better space resolution than acoustic sensors [1,2,3].

Interaction mechanisms between laser radiation and matter can be used to develop sensors of «olfaction». Such sensors can detect and identify chemical elements that are a combination of oil hydrocarbons in seawater or in the object’s underwater environment. Such sensitivity feasibilities in underwater robotics are based on the methods of laser induced breakdown spectroscopy (LIBS) and laser induced fluorescence (LIF) and are discussed in this article.

The LIF method is widely used to discover the ocean by means of aerial vehicles and marine on-board spectrofluorometers [4], but there are only a few cases where such spectrofluorometers are used by underwater vehicles. A LIF device was installed in a Remotely Operated Vehicle (ROV) to provide laser spectroscopy and monitor the ecology of an underwater environment [5]. Raman scattering spectrofluorometers were improved and DORISS (Deep Ocean Raman In Situ Spectrofluorometer) was developed and tested [6]. The modification of the abovementioned spectrofluorometers improved its characteristics in order to investigate gas hydrates at a depth of 6000 m [7]. It is necessary to have a working class ROV for complex measuring, which results in high research costs and expenses. This fact inhibits the growth of common devices for average underwater investigations and the ecological monitoring of the shelf. The development of a small sized spectrofluorometer with low energy consumption is crucial today [8]. Such a spectrofluorometer might be used by an observation class ROV.

The LIF sensor of an underwater vehicle is necessary to measure chlorophyll a, b and c, which are dissolved organic matter of natural origins, mostly originating from the life processes of biological objects (phytoplankton) in the ocean. This sensor also detects dissolved oil hydrocarbons (anthropogenic origin and natural exposure from the bottom) and measures their concentrations. The best way is to use short wavelength radiation for the excitation of the LIF spectrums (for example 4th harmonics Nd:YAG laser, 266 nm wavelength). In this case the spectral regions of the LIF can be separated and conformed to the dissolved oil hydrocarbons, dissolved organic matter, and chlorophyll fluorescence. Moreover, it is possible to identify oil hydrocarbons [8,9]. There is quite a strong absorption of short wavelength radiation in seawater, so the distance to underwater objects under investigation is limited. However, the adaptation of LIF for the anthropomorphic complex of an underwater vehicle can deliver laser radiation to exact areas and register the radiation scattering from objects that are under investigation.

Feasibility studies of LIF were conducted in laboratory experiments by ultra-short laser pulses to measure the concentrations of oil and the oil products in the seawater. The abilities of detection for solutions of crude oil, RMB 30 fuel (ISO–8217) and DMA fuel (ISO–8217) in seawater were investigated by nanosecond and femtosecond LIF. RMB 30 and DMA are the most commonly used fuels for bunkering the vessels. These fuels are usual sources of pollution in seawater.

In spite of the long term research of the LIBS methods [10], there are a few examples of submersible LIBS devices that aim to measure the chemical elements of underwater objects [11]. A LIBS device (ChemiCam module) was installed in a ROV and deep sea in situ tests were done to obtain the chemical composition of seawater and perform a geochemical test of mineral deposits for the first time [12]. The improvement of this method is an ongoing topic of research [13,14,15,16,17,18,19,20,21]. One way to improve this method is to use double pulse and multi pulse laser breakdowns [22,23,24]. The other way is to use ultra-short laser pulses. The new feasibility of femtosecond LIBS for liquids was described in this article.

There is a description of the basic elements of an olfaction device for a ROV in item 2, “Instrument design”, of this article. Also, there is a description of the submersible LIF device that was developed by the authors. The test of the LIF device, which includes laser, detector, and spectrometer, are described.

There is a description of the research laboratory facilities, which was used to discover the feasibilities of the LIF and LIBS methods in item 3, “Materials and methods”, of this article. We suppose that these methods can be improved to develop “olfaction” sensors for underwater vehicles.

A description of the obtained results by ultra-short laser pulses for the LIF and LIBS methods in the laboratory are described in item 4, “Results”. The results of LoD by nanosecond and femtosecond LIF and LIBS were compared.

## 2. Instrument Design

**Figure 1:**
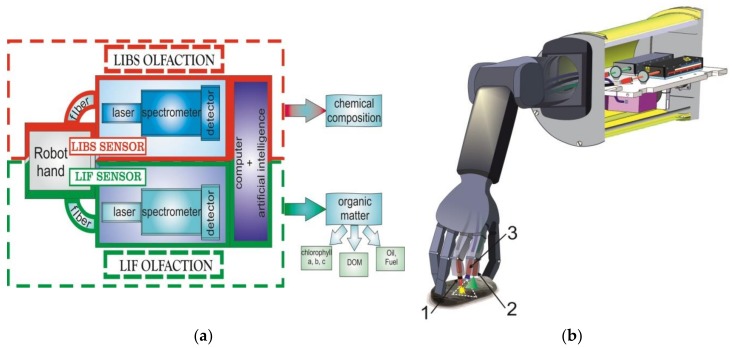
The concept of «”olfaction”; (**a**) Block scheme of “olfaction”; (**b**) Adjustment scheme of Laser Induced Fluorescence (LIF) and Laser Induced Breakdown Spectroscopy (LIBS) for the anthropomorphic complex. 1—laser radiation for LIBS; 2—laser radiation for LIF; 3—lens to focused radiation to spectrofluorometer.

We suppose that a combination of anthropomorphically designed grippers and manipulators (robot hand), LIF and LIBS devices, and artificial intelligence (AI) for underwater robotics advances the abilities of robots and the quality of their underwater activities. The concept of «olfaction» development is shown on Figure 1a. LIF and LIBS sensors have the same structure and consist of LIF and LIBS devices (laser, spectrometer, and detector) and a robot hand. A robot hand will be designed to deliver laser radiation to the object of investigation and then deliver the radiation back to spectrofluorometer. Joint LIF and LIBS sensors with an artificial intelligence software package will provide the «olfaction» of the chemical composition and the different organic matters in the seawater.

The adjustment scheme of the LIF and LIBS sensors, which are being designed for the anthropomorphic complex, is shown in Figure 1b.

**Figure 2:**
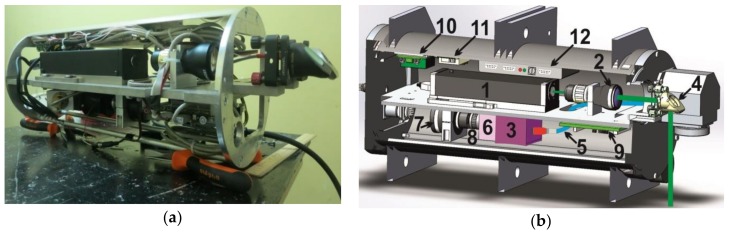
(**a**) Submersible module of the LIF sensor; (**b**) Setup scheme of the submersible module of the LIF device: 1—laser; 2—collimator; 3—spectrometer; 4—rotary mirror of the output radiation; 5—optical multi-core fiber; 6—electro-optical converter; 7—CCD camera Videoscan-285 USB; 8—lens; 9—ITX-N29 processor board; 10—48 to 12 V converter for onboard power supply (laser power supply); 11—48 to 5 V converter (ITX-N29 power supply); 12—computer-controlled laser power supply unit.

Today the robot hand, which will be equipped with an optic fiber, is under development by the authors. A submersible box for the LIF and LIBS sensors was tested (the main elements of the LIF sensor with software) [25]. Figure 2a shows the image of the LIF arrangement with no hermetic box. The setup scheme of the LIF sensors is shown on Figure 2b.

The weight of the LIF sensor is 7 kg and the size is 200×480 mm (diameter and cylinder length). This version is a detachable device for a ROV and can be used without the anthropomorphic complex. The LIF sensor provided LoD values of chlorophyll A 5 µg/L and 15 µg/L for DOM, so this is enough to monitor phytoplankton communities and different types of DOM.

## 3. Materials and Methods

### 3.1. LIF Sensor Development

Solutions of crude oil, RMB-30 fuel, and DMA in seawater were investigated. 1% of the oil solution had been mixed by a magnetic mixer for a long period of time. Complete dissolution was achieved after RMD-30 and the DMA had been being mixed for 24 h. There was no particulate matter in the fuel solution. Particulate matter of crude oil residue was deleted from the samples by filtration. The concentrate was dissolved in seawater in order to be reduced. Samples of seawater were collected from Peter the Great Bay. The LIF spectrums of seawater were measured to obtain a concentration for chlorophyll A and DOM.

Radiation by nanosecond laser pulses was delivered to the cell by optic fiber. Radiation that had been scattered in the solution was delivered back to the spectrofluorometer by optic fiber. Optic fibers of 1.5 m with 600 µm in diameter were chosen to adopt this method for the anthropomorphic underwater vehicle. The distance between the optic fibers’ end-faces, where the radiation was delivered underwater and received from underwater, was 1 cm. Femtosecond laser radiation was delivered exactly to the cell with no optic fiber and the scattering radiation was received in the same way as for the nanosecond pulses.

**Figure 3:**
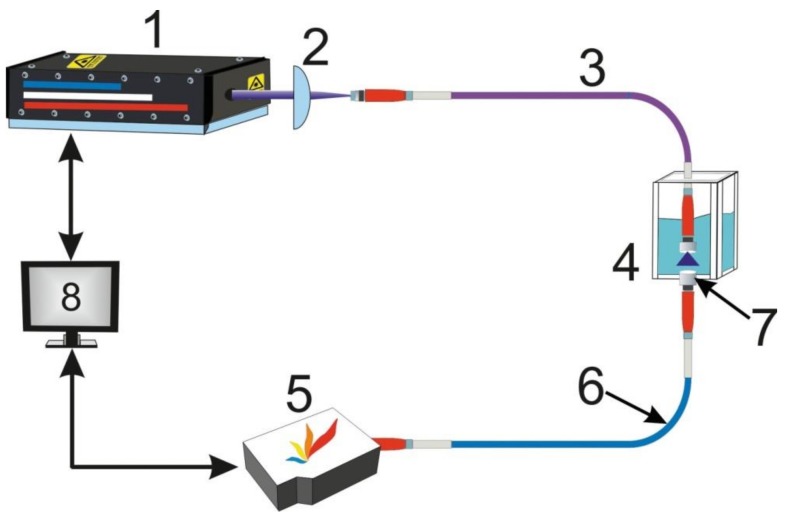
1—Q-Smart 850 (Quantel, Les Ulis, France)/Tsunami + Spitfire 40f-1k-5W (Spectra-Physics, Santa Clara, CA, USA); 2—lens; 3—quartz fiber; 4—cuvette with solution; 5—Maya 2000 Pro spectrometer (Ocean Optics, Largo, FL, USA); 6—fiber with a core diameter of 600 µm; 7—collimator 74-UV (Ocean Optics, Largo, FL, USA); 8—computer.

The scheme of the laboratory setup is presented in Figure 3.

Nanosecond Quantel Q-Smart 850 laser was used and the wavelength was 266 nm, the duration of the laser pulse was 6 ns, the frequency was 20 Hz, and the average power was 20 mW. Femtosecond Tsunami + Spitfire 40f-1k-5W laser complex with third harmonics of radiation (266 nm) was used, the duration of the laser pulse was 60 fs and the frequency was 1 kHz. The average power of the laser pulses was about 25 mW in both cases. Maya 2000 Pro spectrometer (Ocean Optics, Largo, FL, USA) with 600 µm optic fiber and Ocean Optics 74-UV collimator were used to register the spectrums. The spectrums were averaged by three measurements. The total time of measurement of the solution was limited by the burn up of oil products by an ultra violet radiation of high intensity.

### 3.2. LIBS Sensor Development

**Figure 4:**
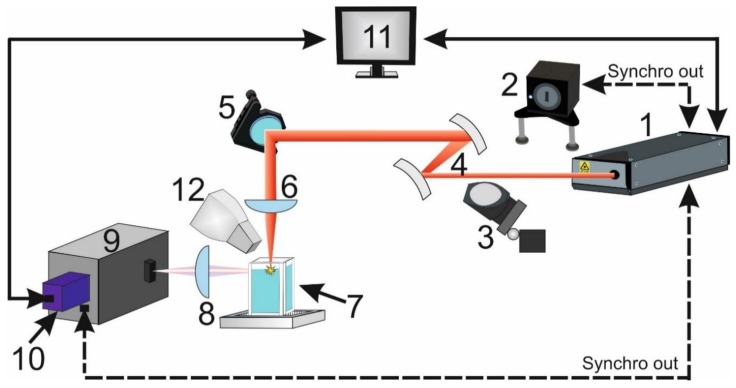
Femtosecond laboratory setup. 1 — Tsunami + Spitfire 40f-1k-5W femtosecond laser complex (Spectra-Physics, Santa Clara, CA, USA); 2—PSCOUT PL-SP-LF (Spectra-Physics, Santa Clara, CA, USA) autocorrelator; 3—folding mirror; 4—1.5× telescope; 5—mirror; 6—KPX094AR.16 (NewPort, Irvine, CA, USA) focusing lens, 7—cuvette with solution, 8—collecting lens, 9—Spectra Pro 2300 spectrograph (Princeton Instruments, Trenton, NJ, USA), 10—ICCD camera (Pi-MAX 3, 1024×1024 pixels, Princeton Instruments, Trenton, NJ, USA), 11—computer, 12—aspirator (for lens protection from water drops).

The improvement of the LIBS method for detecting elements in seawater was achieved through ultrashort laser pulses in the laboratory. The experiment was implemented as follows (Figure 4).

The plasma on the surface of the water sample was formed by femtosecond laser complex (Tsunami + Spitfire 40f-1k-5W(Spectra-Physics, Santa Clara, CA, USA) (1). Ultrashort pulses were about 60 fs duration at a central wavelength of 800 nm with energy of 1 mJ pulse. The repetition rate of pulses could vary within the range of 4–1000 Hz. The pulse duration was monitored using the PSCOUT PL-SP-LF (Spectra-Physics, Santa Clara, CA, USA) autocorrelator (2). A part of the laser radiation was fed to the autocorrelator using a beam splitter mounted on the folding holder (3). The laser radiation was directed through the telescope (4) with a multiplicity of 1.5 to a plane-convex lens with a focal length of 100 mm (KPX094AR.16, NewPort, Irvine, CA, USA). The mirror (5) laser radiation was directed to the KPX094AR.16 (6) with a focal length 100 mm and was focused on the surface of the investigated solution. Samples were poured into a quartz cuvette (7). Radiation from the plasma was directed by lens (8) to the input slit of the spectrometer (Princeton Instruments, Trenton, NJ, USA) (9). Spectrometer was equipped with a 1200 lines/mm grating with a spectral resolution of 2.38 nm/mm. The width of the gap was 50-μm. 16-bit ICCD camera (Pi-MAX 3, 1024×1024 pixels, Princeton Instruments, Trenton, NJ, USA) (10) was used as the radiation detector. The built-in delay generator (Super SYNHRO, Moscow, Russia) provided the optimal log delay time relative to the laser pulse for each sample. The operation of the experimental setup was controlled by a computer (11) via the Ethernet channel. An air aspirator (12) was used to prevent solution spatter from entering the focusing optics and to eliminate the effect of the appearance of an aerosol above the surface of the liquid being examined. All measurements were done with a constant optical configuration and radiation characteristic.

The thermodynamic parameters of the laser plasma induced by the femtosecond laser pulses are quite different from the parameters of plasma, which is generated by a nanosecond laser, and the background continuous radiation of the plasma is relatively weak. Time evolution of continuous and discrete spectrums of plasma are changed when the excitation of the nanosecond laser is changed to femtosecond [13,26].

The following parameters of time resolved registration of spectrums were chosen to reduce the limits of detection of the LIBS method; i.e., signal delay time (t_d_) to laser pulse, and exposure time and integration time (t_g_). While optical breakdowns were being induced, strong continuous radiation appeared and small quantities of the most intensive emission lines were detected on this background. The higher the LoD goes, the stronger and continuous radiation is. Detection should start with a delay of t_d_ from the beginning of the laser breakdown in order to improve the method. The optimal value of t_d_ and t_g_ usually depends on the method of excitation and the type of elements to be investigated. A combination of plasma generation by femtosecond laser pulses with time resolving spectrums registration by the LIBS method might reduce the LoD. The advantages of the LIBS method with laser plasma excitation on the surface of water solutions by single femtosecond pulses were investigated in order to provide a quantity analysis. Also, values of the LoD of the dissolved chemical elements (Al, Ca, Cu, Pb, Zn, Ba, Fe, K, Mg, Na, Sr) were detected. The values of LoD that had been received by a nanosecond laser were compared with the values that had been received by a femtosecond laser.

## 4. Results

### 4.1. LIF

**Figure 5:**
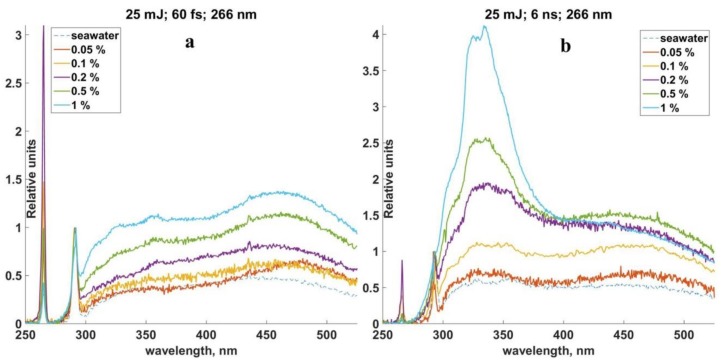
LIF spectrums of crude oil solution in seawater. (**a**) femtosecond LIF; (**b**) nanosecond LIF.

LIF spectrums of crude oil solutions, which were received in laboratory experiments, are shown in Figure 5. Spectrums, which were received by femtosecond laser pulses, are on the left side and spectrums of nanosecond laser pulses are on the right side. Different concentrations of oil are marked by different colors on the diagram (the color of each concentration is explained in the upper side of the figure). Relative values of LIF signal intensity were calibrated to the intensity of the Raman scattering of seawater and were laid off along the vertical axis. Parameters of laser radiation, which were used in this experimental work, are located in the upper part of the diagrams.

Spectral distribution includes two narrow lines (266 nm and 296 nm) and a wide spectral band of fluorescence, which are shown in the diagrams. The first narrow line indicates the elastic scattering of the pumping radiation wavelength after it was suppressed by the filters, the second line is for the Raman scattering of water.

Fluorescence signals of oil and oil products are registered within the range of 300 nm and 550 nm and they are performed by wide bands. Two wide maximums within the range of 320–380 nm and 420–500 nm were observed for the crude oil solution (Figure 5). These maximums can be observed in both cases of LIF excitation. The higher concentration of oil in the seawater, the more intensive the LIF signal grows.

Fluorescence signal of seawater with no oil hydrocarbon is marked by the dashed line and was caused by DOM fluorescence during sample collection. Oil and oil products were dissolved in the seawater, which was collected in the Peter the Great Bay. DOM fluorescence signal is performed in this case as a background, where the LIF signal of oil and oil products can be registered.

**Figure 6:**
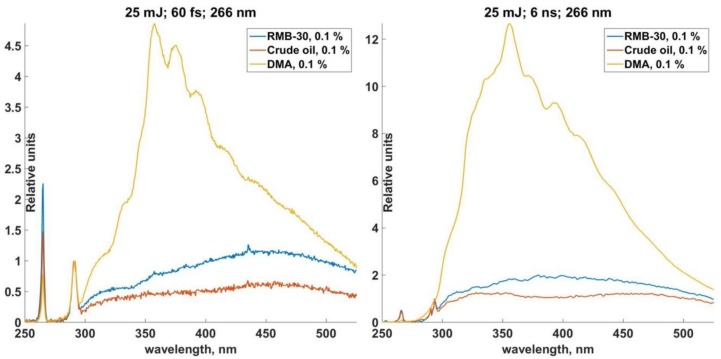
LIF spectrums of crude oil and fuels.

Such spectrums were also received for solutions of RMB-30 and DMA fuels. The range of solution concentration between 1% and 0.05% was also used for RMB-30. The LIF signal from DMA fuel was quite strong, so it was able to provide an investigation of LIF spectrums in the rage of 1–0.0004%. The form of the spectrums with a concentration sample of 0.1% is shown in Figure 6.

LIF spectrums of RMB-30 and DMA are essentially wide lines with maximums. In case of RMB-30 fuel, the maximum was observed in the range of 400–500 nm. The long-wavelength maximum of the oil spectrum was the same. In the case of DMA fuel, the spectrum can be registered in the short waveband of 330–400 nm, which also can be seen in the LIF spectrums of crude oil.

The higher the concentrations of fuel in water, the stronger the intensity of the LIF signal, and this is the same for both the nanosecond and femtosecond laser. Special features of the spectrums, which were received, are as follows:

− the intensity values of the LIF spectrums, which were excited by femtosecond laser, turned out to be small for each solution;
− a relative spectral distribution of the LIF is the same for both the femtosecond and nanosecond LIF;
− specific wide maximums were marked for different fractions of oil and this might be used to identify it; both of these wide maximums are within the spectrum of crude oil.

A value of integral spectral parameter α is offered to be measured to detect concentrations of dissolved oil hydrocarbons and calibrate the LIF spectrums with oil product concentrations; this value is as follows:

(1) $$\alpha =\int_{300}^{525}\left(I\left(\lambda \right)-I_{0}\left(\lambda \right)\right)d\lambda$$

where *I*(*λ*)—the intensity of total signal of LIF on the wavelength *λ*, *I*_0_(*λ*)—LIF signal of seawater without dissolve oil products on the wavelength *λ*. Then this parameter had been calibrated with relevant concentrations of oil and oil products.

**Figure 7:**
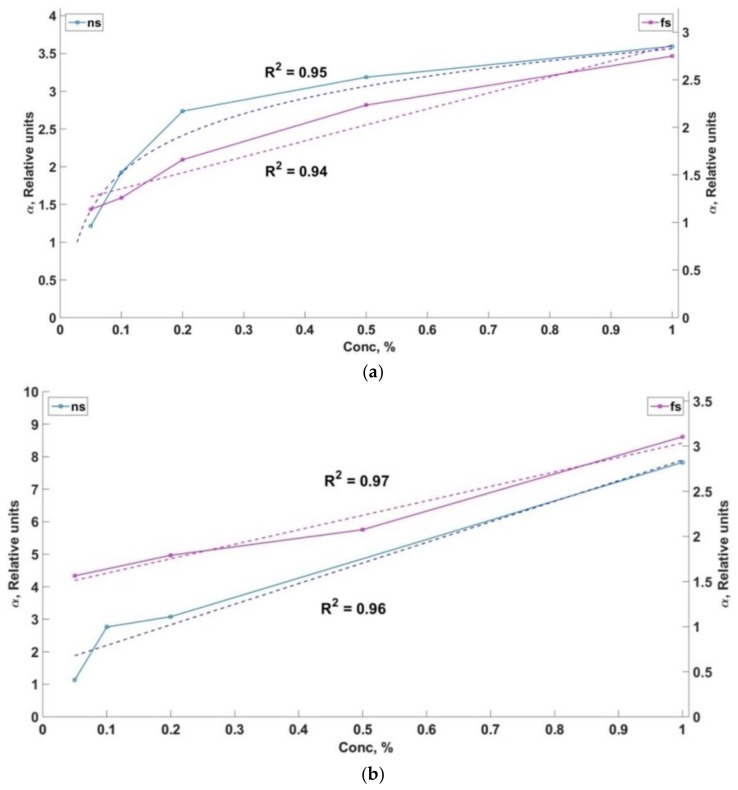

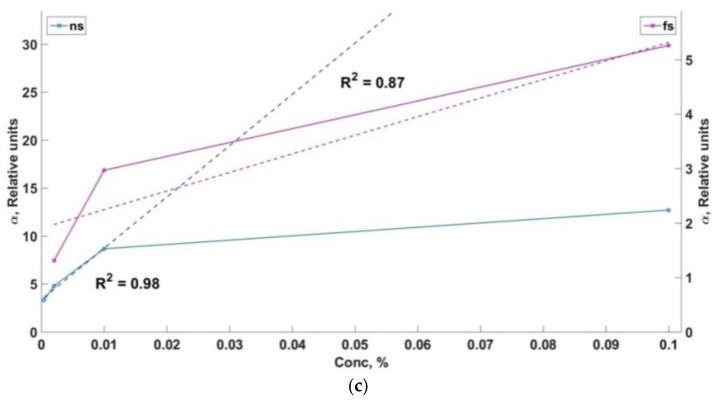
Calibrate functions. (**a**) Calibrate function of crude oil solution; (**b**) Calibrate function of RMB (Residual Marine B)-30; (**c**) Calibrate function of DMA (Distillate Marine).

There are calibration functions of different solutions of oil carbons in seawater in case of the usage of nanosecond and femtosecond laser pulses in Figure 7a–c.

The value of α parameter is laid off on the vertical axis (left one is for nanosecond laser pulses and wright one is for femtosecond laser pulses). The concentration of the oil products solution (%) is shown on the horizontal axis. The coefficient of determination R^2^ corresponds to the approximation function marked by the dash line.

It is important to point out that the calibrate functions are approximated by the linear functions much better in the case of femtosecond excitation than in the case of the nanosecond LIF. In the case of the nanosecond LIF the calibration function is nonlinear for oil and DMA fuel. Linear ranges of the function for α parameter with low concentrations were marked in order to estimate the LoD values. Calibration functions are complicated and they are different for each grade of dissolved oil hydrocarbon. In case of crude oil there is a saturation of the fluorescence signal when the concentration goes higher. The analysis of the experiments shows that it is necessary to calibrate a wide range of concentration values and use the preliminary information about the oil grade in order to use the LIF method for the detection of oil hydrocarbon concentration in seawater.

A regression estimation of experimental data was used and the values of the minimum detectable concentrations were estimated by the criteria of LoD = 3σ_n_/S, where σ_n_ is the standard deviation of LIF signal, S—calibration line slope [27]. The LoD values (for our experiments) are shown in Table 1. It is necessary to mention that all data shows a preliminary order of magnitude estimation.

### 4.2. LIBS

The investigation of the laser plasma dynamics and their necessary calibration was provided by femtosecond laser pulses on the surface of solutions in order to examine the LIBS method of values and the LoD reduction for seawater elements and elements consisting of sediments. Standard samples of water solutions of Al, Ca, Cu, Pb, Zn, Ba, Fe, K, Mg, Na, Sr were used to identify the limits of detection, and the concentrations of the solutions had been changed in the range of 1.00 g/L up to the LoD values.

**Figure 8:**
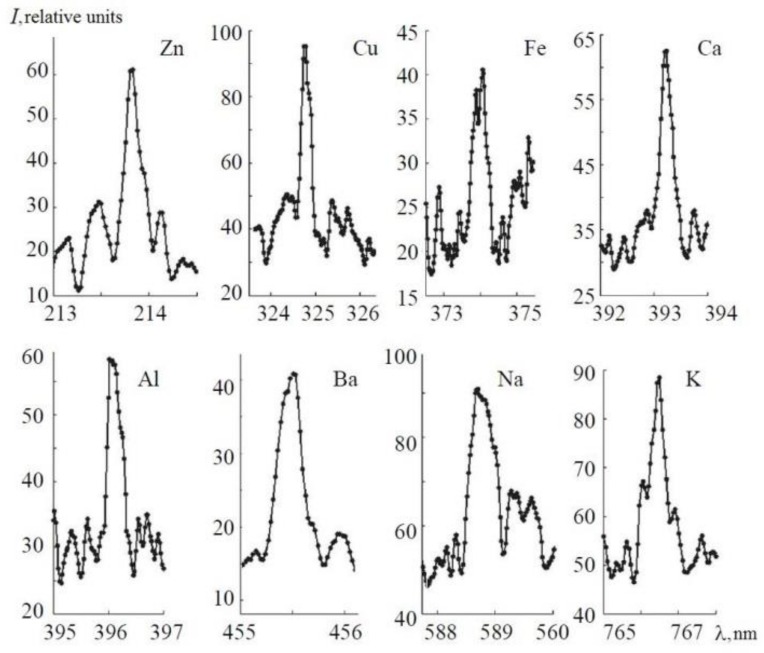
Emission of spectral elements in calibrated solutions by femtosecond laser pulses.

Fragments of emission lines of Zn, Cu, Fe, Ca, Al, Ba, Na and K solutions are shown in Figure 8 in spectral ranges, which were chosen for the investigation of the laser plasma dynamics.

**Figure 9:**
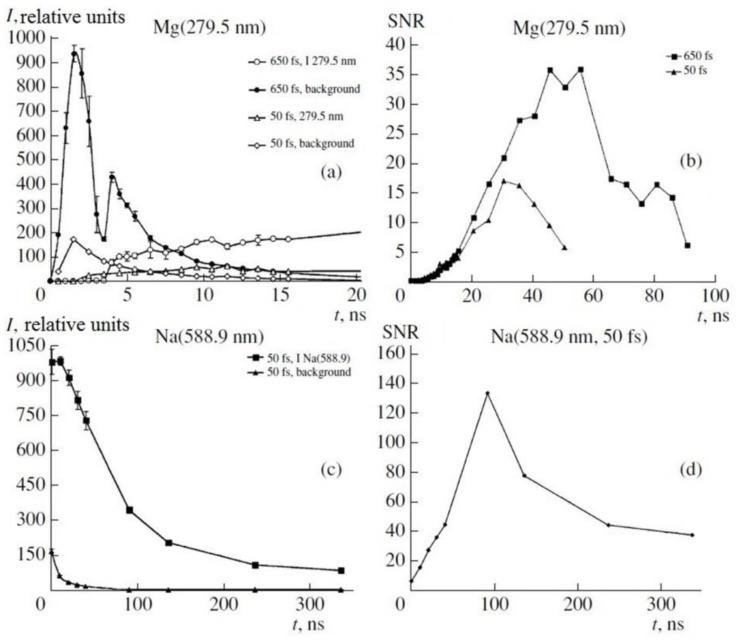
Time dependence of line intensity and background intensity for Mg (279.5 nm) (**a**) and Na (588.9 nm) (**с**) lines; time dependence of contrast K for Mg (279.5 nm) (**b**) and Na (588.9 nm) lines (**d**).

The estimation of the optimal values registration delay to the laser pulse (t_d_) and exposition time (t_g_) was provided for all the elements that we chose and for two of the values of laser pulse durations. Time dependency of emission line intensity of MgI (279.5 nm) and Na line (588.9 nm) are shown in Figure 6. Also, a dependence of the value ratio of the emission line intensity to the intensity of the continuous spectrum in the spectral interval (SNR), which is the most close to the emission line, is also shown. These dependences for Mg are shown in both laser pulse durations of 50 fs and 650 fs (Figure 9a). It is clear that the most intensive background was observed in the first 10 ns when the duration was 50 fs and 650 fs, at the same time intensity the emission line was close to a maximum of 10 ns. Concerning the abovementioned facts, the signal-noise ratio begins to rise rapidly only in the first 10 ns.

The same dependences are shown of Na atoms for a 50 fs duration of the excitation pulses (Figure 9c). So the same dependence was observed, but the SNR maximum was quite a bit longer than Mg and was 100 ns. These results demonstrate the complex dependence of SNR from the beginning of the time registration, excitation pulse duration, and type of element on the background of the continuous plasma radiation. These measurements must be done for each element and optimal values of t_d_, t_g_ must be chosen to receive LoD minimums.

**Figure 10:**
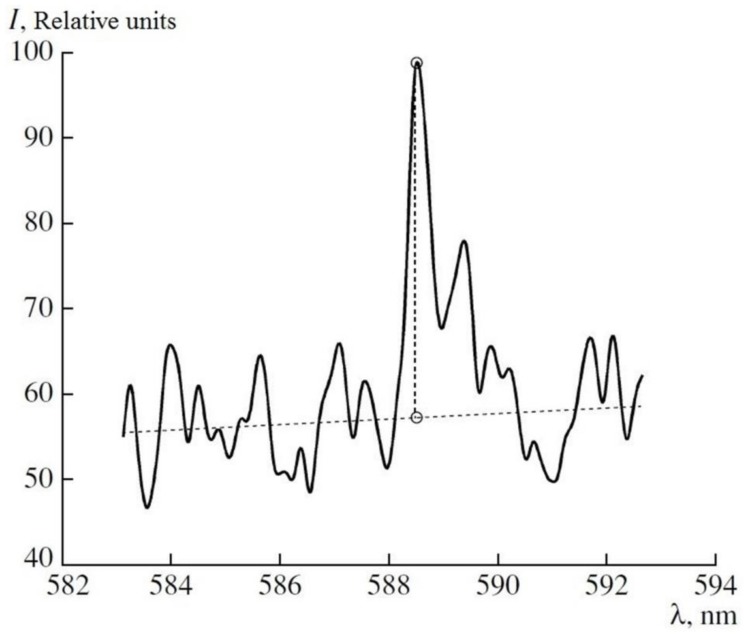
Na doublet in an aqueous NaCl solution with an Na concentration of 4 × 10^−6^ g/L.

The spectrum of the Na atom is shown in Figure 10 and it performs the way to choose the intensity of the emission line and the background intensity of the plasma radiation. The limit of detection of the Na atoms was detected in a water solution of NaCl concerning the SNR time dependence for the Na emission line and the measurement for different concentrations. The LIBS method provided a limit of detection about 1 µg/L concerning the following parameters. A pulse of 650 fs duration, energy of 1 mJ, time of delay t_d_ = 75 ns, and t_g_ = 300 ns were used. The signal was averaged by 800 laser pulses when the radiation frequency was 100 Hz.

Contrary to the case of nanosecond laser excitation (4–15 Hz repetition rate of the Tisapphire femtosecond laser) we observed the generation of stable plasma at the water interface without spraying droplets of liquid on the focusing optics due to the explosive boiling of water in the laser beam. When plasma is produced by 50-fs laser pulses, supercontinuum and filaments are generated in the near-surface layer of water very often.

**Figure 11:**
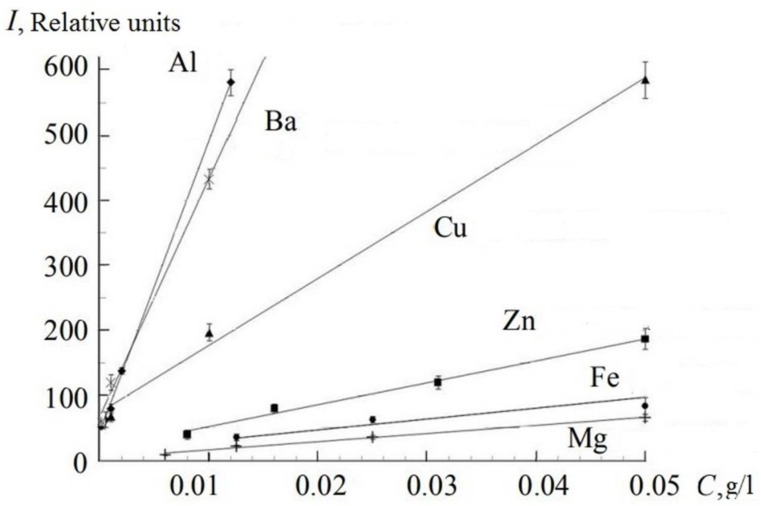
Calibration curves for determining the content of Al, Zn, Cu, Ba, Fe, Mg in water solutions.

Figure 11 shows the calibration curves obtained for Al, Zn, Cu, Ba, Fe, and Mg with concentrations <0.05 g/L. We took the average intensity of the spectral line (see Figure 8), obtained as a result of six measurements, after subtracting the background intensity. All the calibration lines, obtained for concentrations <0.05 g/L, were approximated by linear dependence.

The limits of detection were compared for 11 elements concerning all the abovementioned measurements and the results are shown in Table 2.

The analytical wavelengths of the most sensitive lines of the elements used in this work are indicated in Table 2. The LoD was calculated by the method: LoD = 3σ_n_/S, where σ_n_ is the standard deviation of the background in the pure sample (distilled water) and S is the slope of the calibration curve [27]. Table 2 shows a comparison of the LoDs, which were obtained in this work, with the results from the other studies.

We see that we obtained lower LoDs for Al, Ca, Fe, Ba, K, and Na comparing to values of LoD, which were received by nanosecond LIBS and described in other articles. The LoD for Mg is a little bit higher than in the referred article [28]. However, special pre-processing technology was used in this research work. For Cu, better LoDs were obtained in [29,30], but in this case the plasma was formed in a liquid jet (HCl solution) by emission from an ArF laser with wavelength 193 nm.

Thus, the LoDs obtained by femtosecond time-resolved LIBS showed that the proposed technique is suitable for in situ fast monitoring of the macro composition of seawater (such elements as Ca, Na, Mg, K), with reproducibility of the determinations <10%. The average concentrations in seawater for these elements exceed the LoD values obtained in femtosecond LIBS [31]. Regarding barium, we obtained a value of the same order of magnitude as the average Ba concentration in seawater. This allows us to determine a slight increase in the Ba concentration relative to the average value, for example, at sites of barite deposits or oilfield development.

## 5. Conclusions

The feasibility of laser spectroscopy as sensors of “olfaction” was learned. Different results were obtained for the sensitivity of LIF and LIBS methods by nanosecond and femtosecond laser pulses. Values of LoD were of the same order in the case when organic matters (dissolved crude oil and oil products) in seawater were detected by means of the femtosecond and nanosecond LIF method. Moreover, LoD values were higher for selected dissolved oil products where femtosecond lasers had been used when compared to nanosecond laser induced fluorescence. However, calibration functions are linear for all solutions of oil hydrocarbons in the case of femtosecond laser pulses. Such dependence could be explained by the complex structure and width of the energy levels of the molecule complexes, the specification of fluorescence excitation by laser pulses that are shorter than the time of the transverse relaxation challenging the energy levels of organic complexes in oil and oil products. It is necessary to undertake an additional investigation of laser induced fluorescence for complex oil hydrocarbons that was dissolved in seawater to examine the abovementioned dependences and obtain a more accurate calibration. The values of LoD of oil and DMA fuel are different, so more experiments must be done. However, we have these parameters of the LIF sensor and it is possible to provide the monitoring of environment pollutants caused by DMA fuels. This can be accomplished on the level of the MARPOL convention (threshold limit value is 15 ppm according to MARPOL 73/78, Annex I).

But if femtosecond LIBS is used to measure concentrations of elements, values of LoD are strongly reduced. It is still problematic to adopt a femtosecond LIBS method for the underwater environment because femtosecond laser equipment is quite sophisticated. Also, it is necessary to examine the parameters of femtosecond plasma in the exact underwater environment, with high pressure and a few kilometers of depth. It is supposed that when femtosecond pulses are used to induce laser plasma under high pressure it will provide small values of LoD for elements in the water and sediments. The investigation of processes of laser plasma development on the surface of water solutions shows that femtosecond pulses and methods of time selection for the broadband emission of plasma and emission lines provides a reduction in the LoD values of elements in a water solution. The LIF and LIBS sensors of underwater robotics will improve underwater vehicles for underwater monitoring. Small sized LIF-LIBS devices that do not require strong energy sources can be installed in observation class AUV and ROV. The best way to develop LIF and LIBS sensitivity is to adopt sensors for anthropomorphically complex underwater vehicles. If LIF and LIBS sensors are designed for anthropomorphically complex vehicles it is necessary to provide artificial intelligence of a high level for underwater robotics to control the anthropomorphic complex, movement of the underwater vehicle, and supplement the sensor system.

## Figures and Tables

**Table 1:** LoD values with different concentrations of oil products.

Type of Oil Products	LoD (%), ns	LoD (%), fs
1. Crude oil	0.018	0.047
2. RMB (Residual Marine B)-30	0.027	0.049
3. DM (Distillate Marine) A	0.0003	0.002

**Table 2:** LoD values for trace amounts of chemical elements in water.

**Element**	**Wavelength, Our Work (nm)**	**Gate Delay, Our Work (ns)**	**Repetition Rate (Hz)**	**LOD, Our Data (ppm, fs)**	**Literature Data (ppm, ns)**
AlI	396.1	130	50	0.19	2.7 [32], 10 [33]
Ca I	393.3	55	50	0.01	0.3 [33], 0.94 [15], 1.4 [32], 0.6 [34]
Cu	324.7	92	100	0.78	7 [33], 0.25 [29], 9.6 [35], 12 [36], 0.39 [30], 1.67 [37], 1.7 [32]
Pb	283	130	150	3.8	1.27 [30], 0.13 [29], 13 [36], 12.5 [35], 13.6 [32]
Zn	213.8	57	100	2.5	0.51 [30], 41.6 [32]
Ba I	455.4	72	100	0.08	0.7 [33]
Fe	371.9	160	50	2.6	30 [33], 10.5 [35]
K	766.5	180	100	0.006	0.03 [15], 6.01 [32]
Mg I	279.5	130	150	0.26	1 [32], 0.9 [35], 0.1 [28], 1.0 [33], 0.3 [34]
Na	588.9	210	100	0.0009	0.5 [33], 0.45 [32]
Sr	460.7	130	150	0.44	2.89 [15], 1 [34]
